# Albany County Medical Society

**Published:** 1874-05

**Authors:** F. C. Curtis

**Affiliations:** Secretary


ART. III.—*Albany County Medical Society. Semi-Monthly Meeting, held March 11th*, 1874. Reported by F. C. Curtis, M. D., Secretary.

Dr. John Swinburne, President, in the chair.

About twenty-five members were present.

The name of George W. Papen, M. D., was proposed for membership, and referred.

Dr. James McNaughton made a report as chairman of a committee appointed at a previous meeting to take suitable action in relation to the death of of Drs. John F. Townsend and Peter Van Buren, former members of this Society, who both died in New York City, where they had resided several years, the former on Jan. 8th, 1874, aged 65; the latter, Dec. 5th, 1873.

Dr. S. H. Freeman made a similar report in regard to the death of Dr. J. H. Lasher, who became a member of this Society in 1871, soon after graduating from the Albany Medical College, and died Dec. 12th, 1873, aged 25.

Appropriate resolutions were presented, and, on motion, the reports of both committees were adopted and directed to be entered in full on the minutes of the Society.

Dr. James S. Bailey then read a paper on the subject of "Senile Hypertrophy of the Prostate Gland." He described at length the anatomy of the parts concerned, and gave in detail an account of the general clinical history of the disease under consideration.

The average weight of the healthy gland has been found to be about 4½ drachms, but a weight of less than 7 drachms is not considered abnormal. It becomes enlarged, not necessarily by inflammatory action, but also by increase of tissue, or proper hypertrophy. Either lobe may enlarge separately, or the increase may be uniform of the whole organ.

The effect of this enlargement is to obstruct the flow of urine by changing the size and shape of the urethra passing through it. The canal becomes narrow and tortuous, and the length of the prostatic urethra increases from the normal of 1¼ inches to perhaps 3 inches.

The symptoms of this hypertrophy, coming on in old people, are not marked in the early stage of it. Enlargement may go on for months and years before much change is noted. The constitution of the patient and the character of the enlargement influence this. Then a diminution in force of the ejected stream is observed, and completion is less satisfactory, the bladder not being fully emptied. On account of this same partial retention, the urine becomes ammoniacal and disagreeable. There is a sense of fullness about the part. Pain is developed in the groin and thighs, with an aching, gnawing sensation about the pubes. The bladder walls become hypertrophied and affected with a chronic inflammation. The urethra sympathizes, and there is a smarting soreness, often extending to the glans. Later, there may be inability to retain the urine, which dribbles away and wets the clothing, constituting a most annoying and disagreeable condition. Uremic poisoning may result from backing of the urine up the ureters into the kidneys.

The etiology is obscure. The following are some of the causes to which it has been traced: Horseback riding, calculus, intemperance, cold, gout, rheumatism, external violence, use of catheter, and repeated attacks of gonorrhœa.

The treatment consists in relieving congestion and pain and quieting the disposition to pass water. Hot hip baths are acceptable and beneficial in every stage. Especially where retention exists, these are efficient, and are preparatory to the catheter. Opium may be given in full doses if baths do not relieve. Introduction of the catheter requires great caution, and to facilitate it various forms have been devised. The silver catheter was preferred by Dr. Bailey. The largest size possible should be used, and one having a curve comprising one-fourth of a circle may be most easily introduced in most cases.

Cases were given in illustration. One is as follows:

A strong, powerfully built man, aged 72, by occupation a machinist, took cold, and found difficulty in urinating. Medical assistance was called for on the 7th of July, as he was entirely unable to pass water. A catheter was introduced with difficulty, and three pints of dark colored urine, containing blood, was drawn, which afforded only partial relief to the pain, which was across the loins and down the thighs. It was with difficulty that he changed his position while lying down, or could get in or out of bed. The urine remained tinged with blood until the 22d of July, when catheterization failed to empty the bladder, the blood obstructing the flow through the eyes of the instrument. Dr. John Swinburne was then consulted, and by the use of a No. 11 silver catheter, with large eyes, the thick, bloody urine was evacuated and washed out by means of a fountain syringe, tepid and cold water being used alternately. Upon the next catheterization the urethra seemed shortened more than an inch, and the urine escaped clear, with only an occasional cogula. About this time he manifested symptoms of uremic poisoning; delirium and convulsions occurred. The bladder was washed out twice a day, and the symptoms gradually improved during this time; then his appetite began to fail, and he finally died August 3d.

The following is a report of the autopsy made twenty-four hours after death: Rigor mortis marked; abdomen: liver somewhat enlarged, edges rounded; ductus communis choledochus occluded; gall bladder small, containing a few calculi and a drachm of mucus; pancreas thickened and quite hard on section, but normal in appearance; spleen healthy; right kidney healthy; left kidney —the supra venal capsule enlarged to the size of the kidney itself, presenting on section a granular look and pale color; had the appearance of a morbid growth; kidney itself disorganized, with only two or three pyramids remaining; ureters not affected; bladder walls considerably thickened; half an inch from the mouth of the urethra a longitudinal elevation an inch or more high, composed of the hypertrophied third lobe of the postate gland, so situated as to fall as a valve across the mouth of the urethra; it was congested, was soft and rather friable, and contained two or three perforations; prostate enlarged to two inches antero-posterior diameter, and a little more than two inches in the transverse; urethra normal throughout, except at the external orifice, where there was stricture with dense tissue; alimentary canal normal. Thorax not examined.

The urine was examined July 11th and found alkaline, of low sp. gr., containing abundance of albumen and blood globules. July 25th it was found strongly akaline and ammoniacal, with a slight amonnt of albumen and depositing triple phosphates and phosphate of lime.

Dr. Van Derveer reported the following case of enlarged prostate:

W. H. J., aged 66; good habits; shoemaker by trade; usually enjoyed good health. December, 1873, noticed some trouble in passing his urine; desired to do it often; not a feeling of satisfaction when the act was completed. On the 12th, failed to pass any water, though making frequent attempts. At 10 p. m. he was first seen. He was then in little pain; abdominal tumor well marked, reaching up to the umbilicus. An attempt had been made during the afternoon to pass a gum elastic catheter, but no urine flowed following its use. After injecting the urethra with warm oil, passed with ease a No. 12 silver catheter, and drew off about two quarts of urine The use of the catheter was continued for two weeks, twice daily. After this he passed for about twelve days his urine with comparative ease. At this time he was attacked with acute bronchitis, and it became necessary to employ the catheter two to three times daily to relieve his bladder. A thorough tonic course of treatment was adopted, but he gradually sank and died Feb. 10, 1874. A week previous to death a trace of albumen could be found in urine, but no casts.

Post-mortem twelve hours after death. Head not examined. Organs of thorax in a healthy condition. Abdominal organs healthy, excepting kidneys and bladder. The kidneys were very much enlarged; capsule adherent, and glandular structure much congested. The bladder contained a pint of ammoniacal urine; walls thickened, and mucous coat covered with arched and crossed columns of tissue. The middle lobe of the prostate gland was enlarged in such a manner—upward and forward—that when the bladder became at all full this hypertrophied tissue acted as a valve, preventing the exit of urine, and accounting for his in ability to empty his bladder.

The doctor remarked farther that he preferred the olive pointed, black elastic catheter to any any other in enlarged prostate. By dipping in hot water, bending it to the desired curve, which should be an acute one, and then dipping into cold water, the form is retained. He got this idea from Dr. Gouley, of New York, who in turn obtained it from Sir Henry Thompson.

Dr. McNaughton thought that an expert hand was more important than the particular instrument. He had been pleased with Dr. Bailey's paper, and thought he deserved the thanks of the Society for presenting so interesting a resumé of the subject, with cases. His own experience corresponded closely with the views given in the paper. In regard to filling up of the bladder with blood, cases had fallen under his care, but they are not of frequent occurrence. A large catheter should be introduced, and warm water injected. He had never known this to fail in emptying it.

The President said that he had seen one other case only similar to this one which he saw with Dr. Bailey; with Dr. Vedder, of Schenectady. In this the blood was very dark and very fetid.

Dr. Beckett mentioned a case of filling up of the bladder with blood clot, due to a kick of a horse, the bladder being filled with urine at the time. Nothing could be drawn off until the clot was broken up by injecting water.

After a little general discussion of subjects connected with enlarged prostate, the Society adjourned.

